# High continuous fragility index values for randomised controlled trials investigating medial patellofemoral ligament reconstruction for patellar instability: A systematic review

**DOI:** 10.1002/ksa.12701

**Published:** 2025-05-19

**Authors:** Dalraj Dhillon, Paary Balakumar, Prushoth Vivekanantha, Amit Meena, Shahbaz Malik, Darren de SA

**Affiliations:** ^1^ Michael DeGroote School of Medicine McMaster University Hamilton Ontario Canada; ^2^ Division of Orthopaedic Surgery, Department of Surgery McMaster University Hamilton Ontario Canada; ^3^ Gelenkpunkt – Sports and Joint Surgery, FIFA Medical Centre of Excellence Innsbruk Austria; ^4^ Research Unit for Orthopaedic Sports Medicine and Injury Prevention (OSMI) UMIT Tirol – Private University for Health Sciences and Health Technology Innsbruk Austria; ^5^ Department of Orthopaedics and Trauma Shalby Hospital Jaipur Jaipur India; ^6^ Department of Orthopaedic Surgery Worcestershire Acute Hospitals NHS Trust Worcester UK

**Keywords:** fragility, MPFL, patellar dislocation, RCT

## Abstract

**Purpose:**

To evaluate the statistical fragility of randomised controlled trials (RCTs) investigating medial patellofemoral ligament reconstruction (MPFLR) of patients with patellar instability.

**Methods:**

A search of PubMed, MEDLINE, and EMBASE databases for RCTs investigating surgical management of patients with patellar instability from inception to 25 April 2024. Studies that reported over one significant dichotomous outcome, significant continuous outcome, and/or nonsignificant dichotomous outcome were included for analysis. The fragility index (FI), continuous fragility index (CFI) and reverse fragility index (RFI) were calculated for significant dichotomous outcomes, significant continuous outcomes, and non‐significant dichotomous outcomes, respectively.

**Results:**

Thirteen RCTs were included which reported on a total of 929 patients (64.1% female, mean age of 24.4 years [range: 10–47 years] before any patients were lost to follow‐up. The median FI was 1.0 (interquartile range [IQR], 1; 95% confidence interval [CI], 0.725–2.275; range, 0–4). The number of patients lost to follow‐up at the final time point was greater than the study‐specific FI in 7 out of 8 outcomes from four studies (87.5%). The overall median CFI for included RCTs amongst 31 outcomes from nine studies was 11.7 (IQR, 12.9–95% CI, 8.9–13.9; range 0–30.3). The number of patients lost to follow‐up at the final time point was greater than the study‐specific CFI in thirteen outcomes from six studies (41.9%). The median RFI was 7.0 (IQR, 1.0; 95% CI, 6.5–7.5). The number of participants lost to follow‐up was greater than the study‐specific RFI in a single outcome from one study (16.7%).

**Conclusion:**

This systematic review shows that while FI was low at a median of 1.0, consistent with RCTs in sports medicine, the other indicators of statistical fragility were relatively high, especially CFI (11.7). The level of fragility should be taken into account when assessing practical applicability of RCTs on patellar instability, in combination with other indicators of study rigour.

**Level of Evidence:**

Level I.

**Abbreviations**
CFI: continuous fragility index
CI: confidence interval
FI: fragility index
IQR: interquartile range
MPFL: medial patellofemoral ligament
MPFLR: medial patellofemoral ligament reconstruction
PRISMA: preferred reporting items for systematic reviews and meta‐analyses
PROM: patient reported outcome measure
RCT: randomised controlled trial
RFI: reverse fragility index
R‐AMSTAR: revised assessment of multiple systematic reviews
SD: standard deviation

## INTRODUCTION

The medial patellofemoral ligament (MPFL) is the primary lateral stabilising structure of the patella [22]. Rupture of the MPFL occurs in up to 95% of traumatic or atraumatic primary lateral patellar dislocation and patients with these often face recurrent patellar instability as a result [22]. MPFL tears account for 3% of knee injuries encountered in orthopedic practice and tend to impact young, active, female athletes with the greatest frequency [6, 15, 47]. Nonoperative treatment for MPFL injuries predominantly focuses on immobilisation followed by physiotherapy with a focus on quadriceps strengthening [15]. Despite this, the injury recurrence rate after non‐operative treatments is around 40%–60% which has prompted greater interest in early surgical intervention [15]. Recent literature supports the early surgical intervention in treatment of patellar instability with regard to objective and subjective functional recovery [15, 18]. However, given the wide variety of MPFL reconstruction (MPFLR) surgical techniques and graft choices, there is currently no clear gold standard surgical protocol for MPFLR. While recent randomised controlled trials (RCTs) have been evaluating the effectiveness of these techniques and graft choices, many studies utilise different subjective and objective outcomes. This presents a statistical challenge as studies can contain an appropriate sample size for the primary outcome but not for all measured outcomes. Accordingly, the robustness of RCTs which investigate surgical management of patellar instability must be assessed to better guide treatment protocols for this patient population, robustness being the ability of outcomes to remain unchanged by small difference in results.

The Fragility Index (FI) is one metric which has been developed to assess the strength and robustness of randomised control trials. The FI can be defined as the minimum number of patients whose status would have to change from a non‐event to an event required to turn a statistically significant result to a nonsignificant result. The FI is classically used to assess RCTs which investigate dichotomous outcomes, but the continuous fragility index (CFI) and reverse fragility index (RFI) can be used for continuous outcomes, such as positive patellar apprehension test and persistent patellofemoral instability, but the CFI and RFI can be used for continuous outcome such as patient reported outcome measures [2] and nonsignificant dichotomous outcomes [20], respectively. Lower values in each of these scores represent a measured outcome that could more likely have shown the opposite significance as fewer participant event outcomes are needed to change the significance of that outcome. Various studies investigating the fragility index of RCTs in orthopedic subspecialties have found the FI of orthopedic studies to generally be low, ranging from 2 to 5 [11]. Given this trend, a study investigating the FI of patellar instability RCTs is warranted to assess the robustness of the evidence base. Despite the recent emergence in popularity of MPFLR, no reviews to date have assessed the statistical fragility of RCTs comparing the MPFLR with nonoperative regimens or comparing different techniques of MPFLR. An assessment of the strength and quality of these trials can help guide both clinical practice as well as further trials investigating this topic. Therefore, the aim of this methodological systematic review is to evaluate the robustness and statistical reliability of RCTs investigating MPFLR techniques for patellar instability. It is hypothesised that the results of RCTs investigating surgical management for patellar instability will be statistically fragile [19].

## METHODS

This study followed the PRISMA (Preferred Reporting Items for Systematic Reviews and Meta‐Analyses) and R‐AMSTAR (Revised Assessment of Multiple Systematic Reviews) guidelines for coordinating and reporting systematic reviews [23, 26].

### Search criteria

Embase, MEDLINE, and PubMed were searched from database inception until 25 April 2024. The search looked for randomised controlled trials (RCTs) utilising MPFLR for the treatment of patellar instability. The search term was “(MPFL OR medial patellofemoral ligament) AND reconstruction” (Supporting Information: Table 1). Studies were included if they met the following criteria (1) RCTs comparing MPFLR techniques or MPFLR with conservative management, (2) ≥ 1 statistically significant continuous outcome, statistically significant dichotomous outcome, and/or non‐significant dichotomous outcome, (3) human studies, and (4) studies published in the English language. Exclusion criteria were (1) level II–IV evidence, (2) textbook chapters, (3) conference abstracts, and (4) biomechanical or cadaveric/animal studies. References of included studies and of pertinent review papers were manually searched to ensure all means of study identification were exhausted.

### Screening

Independent and blinded title and abstract screening were conducted by two authors (DD and PB), with conflicts resolved through consensus or consultation with a more senior author (PV). During the full‐text screening stage, studies were independently screened by the initial two authors, and disagreements were resolved in a similar manner.

### Assessment of agreement

Inter‐reviewer agreement was evaluated using Cohen's kappa (*κ*) coefficient statistic for screening. A priori classification was determined using the following criteria: *κ* of 0.91–0.99 was considered to be almost perfect agreement; *κ* of 0.71–0.90 was considered to be substantial agreement; *κ* of 0.61–0.70 was considered to be high agreement; *κ* of 0.41–0.60 was considered to be moderate agreement; *κ* of 0.21–0.40 was considered to be fair agreement; and a *κ* value of 0.20 or less was considered to be no agreement [24].

### Risk of bias assessment

The Cochrane Risk‐of‐Bias Version 2 (RoB2) was used to assess the risk of bias in randomised trials [39]. The tool is structured in five domains of bias including bias arising from the randomisation process, bias due to deviations from the intended interventions, missing outcome data, bias in measurement of the outcome, and bias in selection of the reported result. These are biases that can occur at different stages of a trial including trial design, conduct, and reporting. A series of 'signalling questions' relevant to the risk of bias are used to create a judgement of the study. Integrating answers to these 'signalling questions' results in 'judgements' that can be either 'low', 'some concerns', or 'high' and indicate the risk of bias.

### Data extraction

Data were extracted in an electronic spreadsheet designed a priori (Google Sheets; Google LLC). Extracted data included study characteristics (i.e., authors, year of publication, level of evidence) and demographic data (i.e., sample size, patient age, sex, etc.), follow‐up time, and lost to follow‐up. Treatment comparisons were also recorded. For each significant outcome, sample size and mean outcome values for both groups, as well as *p*‐values were extracted. Outcomes were categorised as continuous or dichotomous. Additionally, outcomes were categorised as either primary or secondary, as well as either subjective or objective. Subjective outcomes were defined as those interpreted by individual patients, such as patient reported outcome measures (PROMs). Objective outcomes were defined as outcomes that are formally assessed including strength, instability rates, and return to sport.

### Fragility calculations

#### Calculation of the FI

The FI was calculated using the method described by a previously established method [19, 44]. A two‐by‐two contingency table was constructed to compare the number of outcomes events to the number of outcome non‐events across both groups. To calculate the FI, one event was added to the group with the fewer number of events while simultaneously subtracting a non‐event from the same group to preserve patient population size. A Fisher's exact test was used to determine the two‐tailed *p*‐value. This process was repeated until the calculated *p*‐value became equal or greater than 0.05, representing a non‐significant outcome. Therefore, the FI was defined as the number of iterations performed until a non‐significant *p*‐value was obtained from the Fisher's exact test [44]. The FI was calculated for every significant dichotomous outcome from each included RCT.

#### Calculation of CFI

To calculate the CFI, the mean outcome values for significant continuous outcomes across both groups were extracted. Those values were used to conduct a Welch *t*‐test. If the resulting *p*‐value was significant (<0.05), the dataset with the higher mean outcome value was identified. The data point in that dataset closer to, but still greater than the mean is moved to the mean of the lower‐mean dataset. Another Welch *t*‐test was conducted. If the resulting *p*‐value was still significant, this process was repeated until the *p*‐value from the Welch *t*‐test became > 0.05, representing a non‐significant difference between the two groups. The CFI was defined as the number of iterations of the Welch *t*‐test performed until a non‐significant *p*‐value was achieved [2, 3].

To calculate the CFI as described above, a full data set is required. However, many RCTs only report the means and standard deviation (SD) of the measured outcomes. To circumvent this, an iterative substitution algorithm has been described which generates a normally distributed candidate dataset for each group that can be used to calculate CFI [3]. The CFI values in this study were all calculated using an online calculator that applies this algorithm and code [3]. To use this calculator, values such as sample size, mean, and standard deviation across both groups in each study were inputted. The tolerance of the calculator is the measure of how similar the simulated dataset must be compared to the RCT outcome values. This was set to 0.01. The number of iterations represents the number of unique datasets simulated to calculate the CFI and was set to 10. The CFI was calculated for each significant continuous outcome from the included RCTs.

#### Calculation of RFI

The RFI was calculated using a similar methodology as the FI but done in the opposite direction. One event was subtracted from the group with a fewer number of events while simultaneously adding a non‐event to the group to preserve the patient population size [20]. A Fisher's exact test was used to calculate the two‐tailed *p*‐value, and this process was repeated until the calculated *p*‐value became significant (<0.05) [20]. Therefore, the RFI is defined as the number of iterations performed until a significant *p*‐value is achieved from the Fisher's exact test. The RFI was calculated for each non‐significant dichotomous outcome in the included RCTs.

### Statistics and outcome reporting

Demographic variables were reported as means with associated SDs, 95% CIs or ranges. For each study, FI, CFI, and RFIs for each outcome at latest follow‐up were averaged. These means were then used to calculate an overall median FI, CFI or RFI, with associated interquartile ranges (IQR). Outcomes were considered significant if *p*‐values were less than or equal to 0.05.

## RESULTS

### Study selection and characteristics

**Figure 1:**
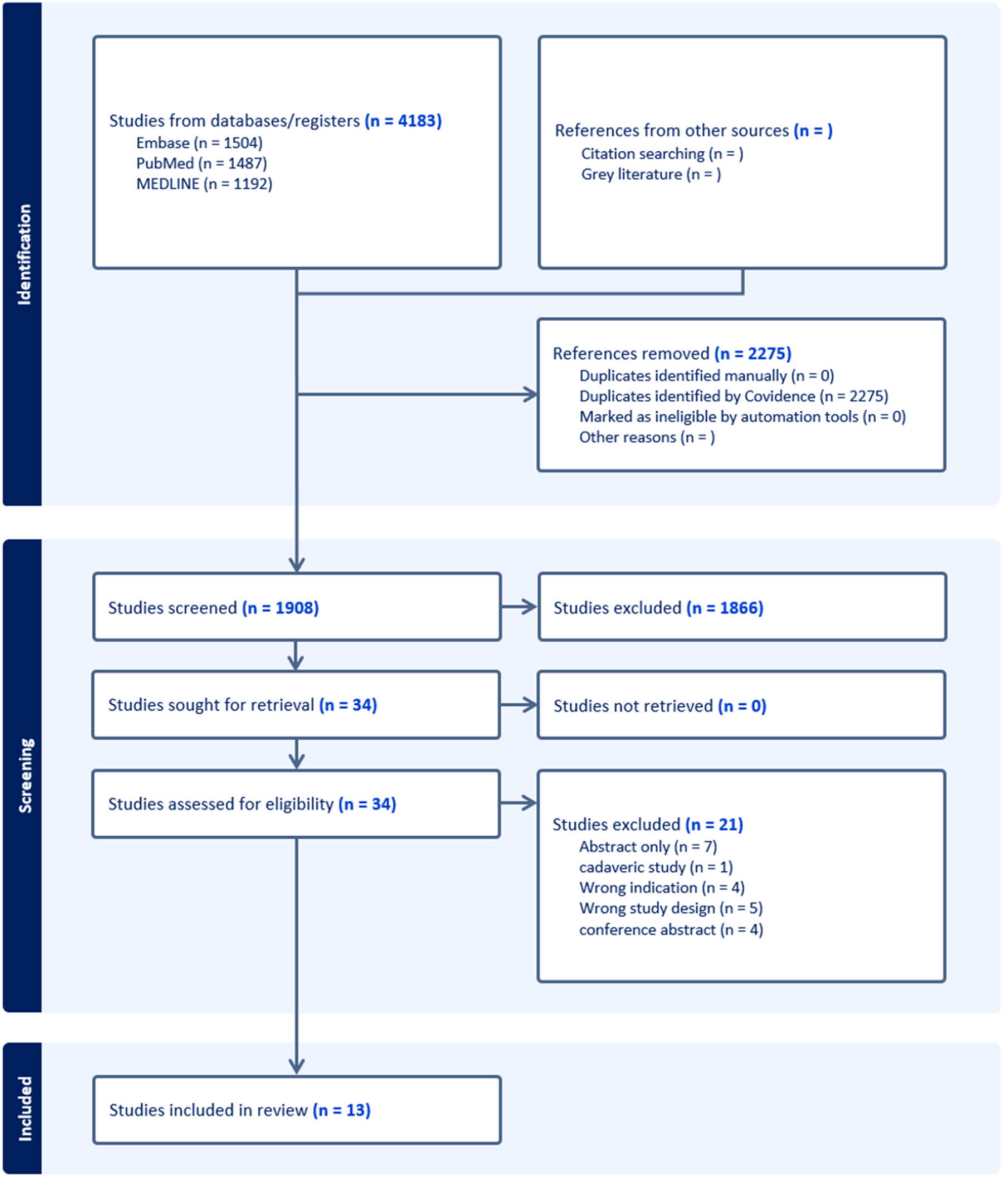
Preferred Reporting Items for Systematic Reviews and Meta‐analyses (PRISMA) flow diagram representing a systematic review analysing statistical fragility of randomised controlled trials (RCTs) investigating medial patellofemoral ligament reconstruction (MPFLR).

A total of 4183 studies across EMBASE, PubMed and MEDLINE were identified. 2275 duplicate studies were removed, resulting in 1908 studies remaining for title and abstract screening. After applying the eligibility criteria, 13 studies were identified and included (Figure 1) [7, 16, 17, 25, 27, 28, 31, 32, 37, 40, 45, 46, 48]. Inter‐reviewer agreement evaluated using Cohen's kappa (*κ*) coefficient showed substantial agreement during the title and abstract screening (*κ* = 0.733 95% CI 0.635–0.832) and full‐text screening (*κ* = 0.762 95% CI 0.568–0.956).

**Figure 2:**
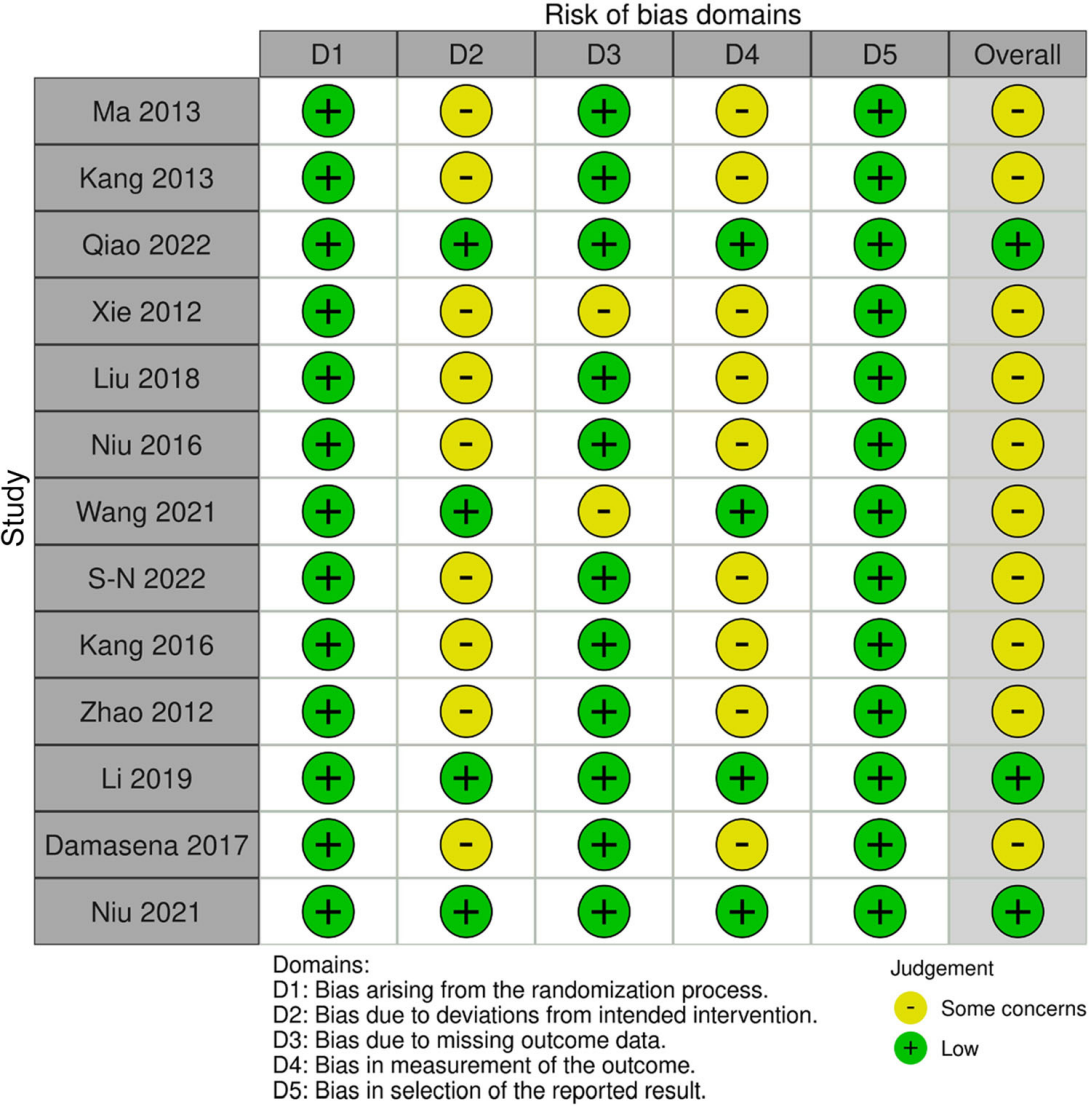
Risk of bias using the risk of bias 2 (RoB2) tool for randomised controlled trials (RCTs) investigating medial patellofemoral ligament reconstruction (MPFLR).

The 13 included studies included a total of 929 patients (64.1% female, mean age of 24.4 years [range: 10–47 years]) before any patients were lost to follow‐up. The mean follow‐up time was 37.8 (SD 16.0) months. Only outcomes that were recorded at the final‐follow up period were included. The mean percentage of lost to follow‐up per study, recorded only at the final follow‐up period, was 10.4% (SD 9.01%). A total of 46 individual outcomes were included and analysed, of which 15 were primary outcomes and 31 were secondary outcomes. Of the 46 outcomes, 21 were objective outcomes and 25 were subjective outcomes (Table 1).The RoB2 assessment showed three studies with 'low' concerns for bias with the remaining ten studies showing 'some concerns of bias'. All thirteen studies showed 'low' concerns in Domain 1: Risk of bias rising from the randomisation process (Figure 2).

**Table 1:** Demographic characteristics.

First author (year of publication)	Journal	Trial comparison	Sample size	Females (%)	Follow‐up time (months)	Lost to follow‐up	Number of outcomes	Number of primary/secondary outcomes	Number of objective/subjective outcomes
Damasena (2017)	*The American Journal of Sports Medicine*	LR + TTT vs. LR + TTT + MPFLR	34	74	60	3	1	0/1	0/1
Kang (2016)	*The Knee*	SB vs. AV	53	53	33	5	2	1/1	2/0
Kang (2013)	*The American Journal of Sports Medicine*	y‐graft vs. c‐graft	82	61	24	3	2	1/1	0/2
Li (2019)	*Arthroscopy: The Journal of Arthroscopic & Related Surgery*	SBTT vs. DAAR	91	62	40	3	5	2/3	3/2
Liu (2018)	*Knee Surgery, Sports Traumatology, Arthroscopy*	MPFLR + LRR vs. MPFLR + LRP	59	63	24	0	2	1/1	1/1
Ma (2013)	*Arthroscopy: The Journal of Arthroscopic & Related Surgery*	MRP vs. MPFLR	63	65	40	7	1	0/1	1/0
Niu (2016)	*International Journal of Clinical and Experimental Medicine*	MRP vs. DBA‐MPFLR	44	57	48	10	5	2/3	3/2
Niu (2021)	*The Journal of Knee Surgery*	DS vs. QS	80	66	40	4	6	0/6	2/4
Qiao (2022)	*Arthroscopy: The Journal of Arthroscopic & Related Surgery*	ST‐MPFLR vs. DT‐MPFLR	90	66	38	4	4	1/3	1/3
Straume‐Næsheim (2022)	*Knee Surgery, Sports Traumatology, Arthroscopy*	AR vs. MPFLR + AR	61	72	12	2	2	1/1	2/0
Wang (2021)	*International Journal of General Medicine*	MPFLR vs. MPFLR + LPRR	87	57	12	29	2	1/1	0/2
Xie (2012)	*The American Journal of Sports Medicine*	MPFLR vs. MPFPLR + polyester Suture Augmentation	85	NR	60	15	10	4/6	6/4
Zhao (2012)	*The American Journal of Sports Medicine*	MPFLR vs. A‐MRP	100	73	60	12	4	0/4	0/4

Abbreviations: A‐MRP, arthroscopic medial retinaculum plasty; AGG, autologous gracilis graft; AR, active rehabilitation; AV, arthroscopy‐view; BTF, bone‐tissue fixation; DAAR, double‐anchor anatomic reconstruction; DBA, double‐bundle anatomical; DS, double strand; DT, double‐tunnel; FLA, fascia lata allograft; LPRR, lateral patellar retinaculum release; LR, lateral release; LRP, lateral retinaculum plasty; LRR, lateral retinacular release; MPFLR, medial patellofemoral ligament reconstruction; MRP, medial retinaculum plasty; NR, not reported; QS, quadruple strand; SB, self‐balance; SBTT, single‐bundle transpatellar tunnel; ST, single‐tunnel; STF, soft‐tissue fixation; TTT, tibial tubercle transfer.

### Fragility index

**Figure 3:**
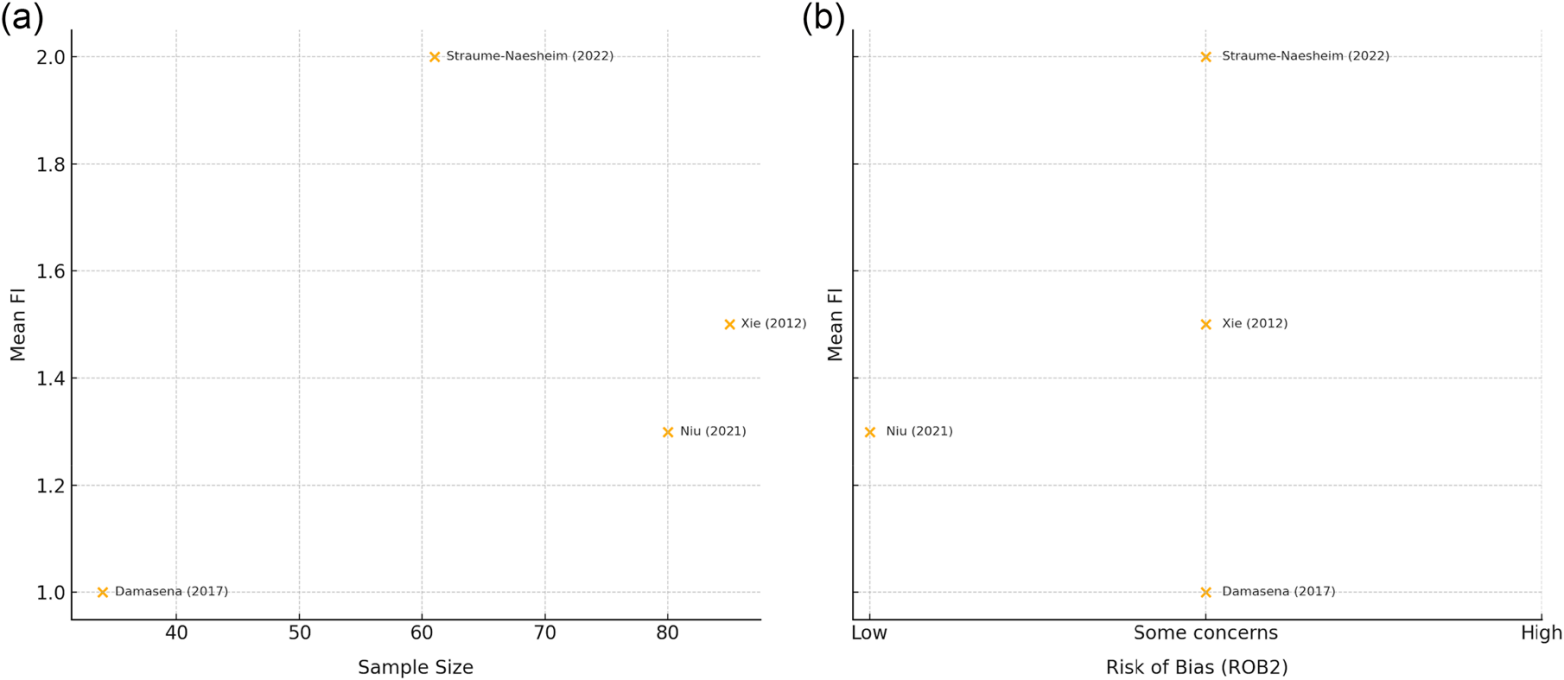
Scatter plot of mean fragility index (FI) values versus a) sample size (left) and b) risk of bias (ROB) (right) via the ROB2 tool.

The overall median FI for included RCTs, calculated across eight outcomes from four studies [7, 32, 40, 48] was 1.0 (IQR, 1; 95% CI, 0.725–2.275; range, 0–4) (Table 2). One primary dichotomous outcome from one study was significant, with an FI of 4 (Table 2) [40]. Seven secondary, significant dichotomous outcomes from four studies were identified with a median FI of 1.0 (IQR, 0.5; 95% CI, 0.670–1.616; range, 0–2) (Table 2) [7, 32, 40, 46]. Four objective significant dichotomous outcomes were identified with a median FI of 1.5 (IQR, 1.75; 95% CI, 0.301–3.199; range 0–4). Four subjective significant dichotomous outcomes were also identified with a median FI of 1 (IQR, 0.25; 95% CI, 0.826–1.674; range, 1–2). Seven of eight outcomes from four studies (87.5%) had a higher number of patients lost to follow‐up at the final follow‐up period compared to the study‐specific FI. Of these studies, the number of patients lost to follow‐up on average was 5.6 (95% CI, 1.839–9.304) more than the study‐specific FI [7, 32, 40, 46]. (Table 2). Two significant dichotomous outcomes from two studies assessed persistent apprehension, with a median FI of 1.0 (IQR, 1; 95% CI, −0.386 – 2.386) [40, 46]. Two significant dichotomous outcomes from two studies assessed persistent patellar translation and instability, with a median FI of 2.5 (IQR, 1.5; 95% CI, 0.421–4.579) [40, 46]. Four significant dichotomous outcomes from two studies assessed patient‐reported functional outcomes, with a median FI of 1.0 (IQR, 0.25; 95% CI, 0.826–1.674) **(**Table 2) [40, 46]. Scatterplots plotting the mean FI relative to sample size and risk of bias are found in Figure 3a,b.

**Table 2:** Fragility values.

Fragility Index (FI)							
First author	Number of outcomes	Type of outcomes (*p*‐value)	Mean FI	Mean FI ‐ primary	Mean FI ‐ secondary	Mean FI ‐ objective	Mean FI ‐ subjective
Damasena (2017)	1	Patient satisfaction (*p* = 0.04)	1	No significant primary outcomes	1	No significant objective outcomes	1
Niu (2021)	3	Subjective Improvement in symptoms (*p* = 0.039) Return to sport (*p* = 0.012) Crosby‐Insall score (*p* = 0.039)	1.3	No significant primary outcomes	1.3	No significant objective outcomes	1.3
Straume‐Naesheim (2022)	2	Persistent patellofemoral instability (*p* = 0.005) Positive apprehension after 12 months (*p* = 0.021)	2	4	No significant secondary outcomes	4	No significant subjective outcomes
Xie (2012)	2	Positive patellar apprehension test (*p* = 0.02) Normal lateral patellar translation glide (*p* = 0.02)	1.5	No significant primary outcomes	1.5	1.5	No significant subjective outcomes

Continuous Fragility Index (CFI)							
First Author	Number of outcomes	Type of outcomes	Mean CFI	Mean CFI ‐ primary	Mean CFI ‐ secondary	Mean CFI ‐ objective	Mean CFI ‐ subjective
Kang (2013)	2	Lysholm score (*p* = 0.001) Kujala score (*p* = 0.001)	10.8	9.8	11.7	No significant objective outcomes	10.8
Li (2019)	3	Patellar tilt angle (*p* = 0.03) Kujala score (*p* < 0.001) IKDC score (*p* = 0.003)	12.2	18.3	0	0	18.3
Liu (2018)	2	Patellar tilt angle (*p* < 0.05) Kujala (*p* < 0.05)	12.4	9.2	15.5	15.5	9.2
Niu (2016)	5	Patellar congruence angle (*p* = 0.008) Patellar tilt angle (*p* = 0.007) Patellar lateral shift (*p* = 0.004) Lysholm score (*p* < 0.001) Kujala score (*p* < 0.001)	9.5	18.2 CFI of Lysholm Score omitted as it is infinite	6.5	6.5	18.2
Niu (2021)	3	Patellar congruence angle (*p* = 0.03) Patellar tilt angle (*p* = 0.02) Kujala score (*p* = 0.01)	5.1	No significant primary outcomes	5.1	4.1	7.1
Qiao (2022)	3	Lysholm score (*p* = 0.03) KOOS symptom score (*p* = 0.02) KOOS Knee‐related QoL (*p* = 0.04)	3.2	No significant primary outcomes	3.2	No significant objective outcomes	3.2
Wang (2021)	2	Lysholm score (*p* = 0.003) IKDC score (*p* = 0.002)	9.0	8.2	9.7	No significant objective outcomes	9.0
Xie (2012)	8	Congruence angle (*p* < 0.001) Lateral patellar angle *p* < 0.001) Patellar tilt angle (*p* < 0.001) Lateral patellar translation (*p* < 0.001) IKDC score (*p* < 0.001) Lysholm score (*p* < 0.001) Kujala score (*p* < 0.001) Tegner (*p* < 0.001)	18.3	19.3	17.3	17.3	19.3
Zhao (2012)	4	IKDC score (*p* < 0.001) Lysholm score (*p* < 0.001) Kujala score (*p* < 0.001) Tegner score (*p* < 0.001)	25.5	No significant primary outcomes	25.5	No significant objective outcomes	25.5

Reverse Fragility Index (RFI)							
First author	Number of outcomes	Type of outcomes (*p*‐value)	Mean RFI	Mean RFI ‐ primary	Mean RFI ‐ secondary	Mean RFI ‐ objective	Mean RFI ‐ subjective
Kang (2016)	2	Apprehension sign (*p* = 0.50)	7.5	8	7.0	7.5	No nonsignificant subjective outcomes
Li (2019)	2	Lateral patellar translation grade (*p* = 0.26) Firm end point in lateral translation (*p* = 0.73)	6.5	No nonsignificant primary outcomes	6.5	6.5	No nonsignificant subjective outcomes
Ma (2013)	1	Negative apprehension test (*p* = 0.17)	4	No nonsignificant primary outcomes	4.0	4	No nonsignificant secondary outcomes
Qiao (2022)	1	Clinical failure of MPFLR (*p* = 0.35)	6	6	No nonsignificant secondary outcomes	6	No nonsignificant secondary outcomes

Abbreviations: IKDC, Knee Injury and Osteoarthritis Outcome Score; KOOS, International Knee Documentation Committee; QoL, quality of life.

### CFI

**Figure 4:**
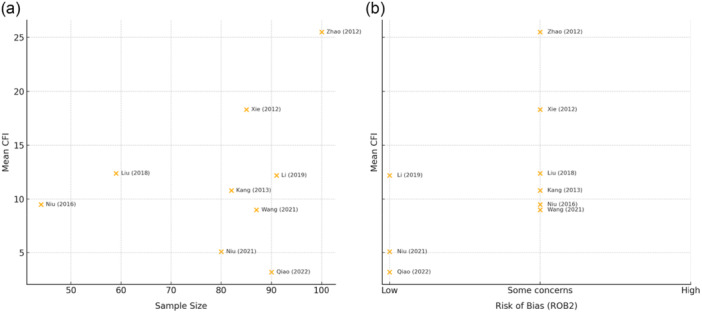
Scatter plot of mean continuous fragility index (CFI) values versus a) sample size (left) and b) risk of bias (ROB) (right) via the ROB2 tool.

The overall median CFI for included RCTs amongst 31 outcomes from nine studies [16, 25, 27, 31, 32, 37, 45, 46, 48] was 11.7 (IQR, 12.9, 95% CI, 8.9–13.9; range 0–30.3) (Table 2). One included study featured an outcome with an infinite CFI indicating that significance could not be reversed even if all data points were to be changed over to the other group [40]. This outcome was excluded from calculations. Fifteen primary continuous outcomes from seven studies [16, 25, 27, 31, 32, 45, 46] were significant, with a median CFI of 11.7 (IQR, 9.75; 95% CI, 10.8–15.3). Seventeen secondary continuous outcomes from six studies [25, 27, 31, 37, 46, 48] were significant, with a median CFI of 15.5 (IQR, 15.8, 95% CI, 2.0–30.3). Eleven objective continuous outcomes from five studies [25, 27, 31, 32, 46] were significant, with a median CFI of 7.0 (IQR, 8.6, 95% CI, 7.5–12.9). Twenty subjective continuous outcomes from nine studies [16, 25, 27, 31, 32, 37, 45, 46, 48] were significant, with a CFI of 14.5 (IQR, 11.6, 95% CI, 11.9–18.0). The number of patients lost to follow‐up at the final follow‐up period was more than the study‐specific CFI in thirteen outcomes from six studies (41.9%) [25, 31, 32, 37, 45, 46]. Of these, the number of patients lost to follow‐up was, on average, 5.4 (95% CI, 3.1–7.7) more than the study‐specific CFI (Table 2). The median CFI was 15.7 (IQR, 10.9; 95% CI, 13.5–19.0) for eighteen outcomes assessing various measures of knee or patient postoperative function from nine studies [16, 25, 27, 31, 32, 37, 45, 46, 48] and 7.0 (IQR, 8.6; 95% CI, 7.5–12.9) amongst eleven outcomes which anatomical positioning of the patella from six studies [25, 27, 32, 37, 46, 48]. Scatterplots plotting the mean CFI relative to sample size and risk of bias are found in Figure 4a,b.

### RFI

**Figure 5:**
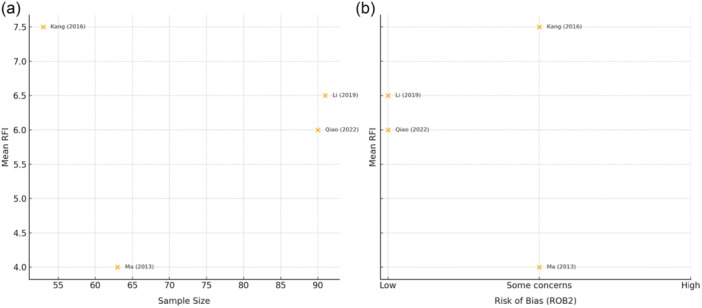
Scatter plot of mean reverse fragility index (RFI) values versus a) sample size (left) and b) risk of bias (ROB) (right) via the ROB2 tool.

The overall median RFI for included RCTs amongst six outcomes from four studies [17, 25, 28, 37] was 6.5 (IQR, 1.0; 95% CI, 5.9–6.8; range 4.0–8.0) (Table 2). Two primary nonsignificant dichotomous outcomes from two studies [17, 37] were identified, with a median RFI of 7.0 (IQR, 1.0; 95% CI, 6.5–7.5). Four secondary nonsignificant dichotomous outcomes from three studies [17, 25, 28] were identified, with a median RFI of 6.5 (IQR, 1.5 95% CI, 5.5–6.5). All six of the nonsignificant dichotomous outcomes were categorised as objective outcomes and assessed measures of instability. The number of patients lost to follow‐up at the final follow‐up period was more than the study‐specific RFI in a single outcome from one study (16.7%) [28]. The number of patients lost to follow‐up was three more than the study‐specific RFI for this study. Scatterplots plotting the mean RFI relative to sample size and risk of bias are found in Figure 5a,b.

## DISCUSSION

In this methodological systematic review of RCTs investigating MPFLR for patellar instability, the primary finding of this study was that the median FI, CFI and RFI were 1.0, 11.7 and 6.5, respectively. These values represent the number of patients required to reverse the statistical significance of the outcomes which were assessed. For the assessed outcomes used to calculate FI, CFI and RFI, a greater number of patients were lost to follow‐up than were needed to reverse the significance of the given outcome in 75%, 41.9% and 16.7% of RCTs, respectively. Like other reviews that have investigated the concept of fragility, FI was the lowest with CFI and RFI being more robust. This indicates significant dichotomous results reported in RCTs on MPFLR are very fragile as only a median of one patient's result needed to be reversed for the outcome to be nonsignificant. On the other hand, the significant continuous outcomes were relatively less fragile as the median number of patients needed to switch treatment groups for a result to become insignificant was higher is 11.7. The fragility of nonsignificant dichotomous outcomes was greater than for significant continuous outcomes, but lower than significant dichotomous outcomes with a median of 6.5 patients needing to change groups in order to reverse the results of these outcomes to significance.

By large, the results of this review suggest that RCTs investigating MPFLR are comparable robustness to when measured by statistical fragility to other trials in various domains of sports medicine and orthopaedics. Previously reported FI values were lower than the FI values reported by trials investigating the orthopedic subspecialties of arthroplasty, foot and ankle, oncology, paediatrics, shoulder, spine, sports and arthroscopy and trauma [1, 5, 8, 9, 10, 11, 14, 19, 21, 35, 36] with the median FI across these subspecialties ranging from 2–9.6. The median CFI in this study was greater than the CFI reported by trials in foot and ankle [1, 13] and shoulder surgery [2, 8] (median ranging from 2.4–9). A review of fragility of orthopedic spine trials [34] however reported a median CFI of 14 which was greater than this study. The median RFI value in this study is also comparable to previous reviews in arthroplasty [12, 14, 38] (median ranging from 5–7), but greater than the RFI reported by one review in foot and ankle surgery [36] (median of 4). These results support the notion that the robustness of literature evaluating MPFLR is comparable to findings reported in established studies in other orthopedic domains. As noted in previous analyses of statistical fragility in orthopaedics and sports medicine, a key reason for this is the difficulty in patient recruitment as sample sizes are often just large enough to reach significance [5, 9, 19, 21, 35, 36]. FI is also known to be positively correlated with event numbers and negatively correlated with p‐values [43]. This also suggests that RCTs evaluating orthopedic and sports medicine practices need more participants. Additionally, this suggests that the techniques/procedures compared in these RCTs have very similar outcomes and are difficult to differentiate based on the variables used to evaluate their effectiveness.

In the 13 studies included in this review, many of the significant outcomes were not the primary outcomes being assessed. Amongst the 40 significant outcomes assessed (FI and CFI), 27 were secondary outcomes, while only 13 were primary. Most primary outcomes in these studies were nonsignificant continuous outcomes and were therefore not included in the FI, CFI or RFI calculations. This is significant as most studies are powered to their primary outcome and not to secondary outcomes [42]. The significant primary outcomes studied in this review include the persistent patellofemoral instability, patellar tilt angle and the Kujala score. Studies should be powered for all of the assessed variables, primary and secondary, when possible as many studies only find significant differences in secondary outcomes which could have more robust findings if studies were powered correctly.

While these fragility values are important to consider when evaluating the robustness of orthopedic literature, their interpretation must consider the limitations that exist with these metrics. Namely, these values may not accurately reflect the overall fragility of a study. The factors which influence the statistical significance of a study are very nuanced and include parameters such as sample size, loss to follow‐up, patients excluded from analysis and bias [33]. Given that statistical fragility does not account for the nuances which influence the significance of the papers, its representation of the robustness of a study is limited. Furthermore, there is yet to be a consensus on the practical application of fragility measures [33]. Thus given the limitations of this fragility analysis, the presented results should not be interpreted as the sole proxy of robustness, but rather as contributing to a holistic evaluation of the robustness of orthopedic literature [33]. With the CFI value being much higher than that of FI, it is possible that FI skews readers' perspectives to think that RCTs may be more fragile than they actually are. Furthermore, there were by far more values for CFI than RFI and FI. This further reinforces that CFI should be a mainstay in evaluating RCT robustness.

It is also important to identify methods to improve fragility. Previous literature has shown that the key factors associated with increasing FI in surgical research are decreasing *p*‐values and increasing sample sizes and event numbers [43]. However, this is a major challenge in surgical research as including more patients is difficult [4, 30]. To increase sample sizes in surgical research, multi‐centre collaborations, innovative trial design and robust a priori sample size calculations should be considered. Participants being lost to follow‐up is also a major factor of smaller sample sizes in surgical studies. Some strategies to minimise loss to follow up include clear communication of study requirements, an expectation to participants and monetary incentives that only pay‐out after follow‐ups [29]. Future RCT committees are advised to carefully consider these principles prior to conducting these trials.

The results of this study may be applied to the clinical practice of orthopedic surgeons. Namely, the FI, CFI, RFI of this study should be used in conjunction with measures of statistical significance to develop a more holistic assessment of the quality of RCTs which are being used to augment clinical practice. Furthermore, the FI provides clinicians with an additional tool which may be conveniently used to assess treatment impact on patients [41]. In addition to the clinical application of our results, it is recommended that researchers consider the following in subsequent analyses of robustness and quality of orthopedic research. First, the development of improved methods for the assessment of robustness for studies using parameters which assess other contributors to statistical fragility such as demographics, study design, etc. Second, it is important to consider powering of studies for multiple outcomes or separate statistical analyses with appropriate powering for each unique outcome in order to improve the fragility of trials. Finally, the inclusion of CFI, FI and RFI is recommended in the planning and sample size assessment of future orthopedic RCTs, as well as in the interpretation of RCTs.

The strengths of this present study include the breadth of studies screened for inclusion as three medical databases were used. Additionally, independent and blinded authors were used to screen and extract the data. Agreement between authors showed substantial significance. As this is a review of RCTs, the included studies were of high quality (Level I) and RoB2 analysis showed three studies with 'low' concern of bias and ten studies with 'some concerns' of bias. Additionally, this study included a thorough statistical analysis including FI, CFI, and RFI whereas many other orthopedic‐focused studies only focus on FI thereby limiting the review scope to include only significant dichotomous outcomes. This study additionally analysed significant continuous and non‐significant dichotomous outcomes.

This review features some limitations to be considered. First, our analysis of fragility focuses exclusively on statistical measures and does not address potential biases or variation in designs which may also affect results. Furthermore, our inclusion of RCTs with only dichotomous or continuous outcomes may have eliminated trials with complicated or nuanced results, reducing the study's comprehensiveness. Furthermore, the RCTs included were heterogeneous and the use of FI, CFI and RFI comes with inherent limitations as they oversimplify trial robustness, particularly when applied to heterogeneous datasets. Finally, as follow‐up periods varied from 12–60 months, only the final time point was included which could have biased the findings. The results of this fragility analysis should be used alongside additional factors when determining the robustness of a given study such as patient population, recruitment strategies, and blinding.

## CONCLUSION

This systematic review shows that while FI was low at a median of 1.0, consistent with RCTs in sports medicine, the other indicators of statistical fragility were relatively high, especially CFI (11.7). The level of fragility should be taken into account when assessing practical applicability of RCTs on patellar instability, in combination with other indicators of study rigour.

## AUTHOR CONTRIBUTIONS

**Dalraj Dhillon**: screening; extraction; writing. **Paary Balakumar**: screening; extraction; writing. **Prushoth Vivekanantha**: writing; editing; supervision; idea conceptualisation. **Amit Meena**: writing; editing. **Shahbaz Malik**: writing; editing. **Darren de SA**: writing; editing; supervision.

## CONFLICT OF INTEREST STATEMENT

The authors declare no conflicts of interest.

## ETHICS STATEMENT

There are no relevant ethical disclosures pertaining to research involving human participants and/or animals, and informed consent was not necessary to develop this manuscript.

## Supporting information

Supporting information.

## Data Availability

Data may be made available upon reasonable request at prushoth.vivekanantha@medportal.ca.
