# Supplementary material for: High continuous fragility index values for randomised controlled trials investigating medial patellofemoral ligament reconstruction for patellar instability: A systematic review
Source: Knee Surg Sports Traumatol Arthrosc. 2025 May 19;34(2):494–508. doi: 10.1002/ksa.12701 (PMC12850585; doi:10.1002/ksa.12701)
Supplement: Supplementary file 1 — Supporting information. [file KSA-34-494-s001.docx]

**SUPPLEMENTARY DIGITAL MATERIAL**

**Supplementary Table 1.** Search Criteria

| **Search Criteria** |
| --- |
| 1. MPFL OR medial patellofemoral ligament |
| 1. Reconstruction |
| 1. #1 AND #2 |
